# Analysis of differential gene expression profile identifies novel biomarkers for breast cancer

**DOI:** 10.18632/oncotarget.23061

**Published:** 2017-12-08

**Authors:** Yunbao Pan, Guohong Liu, Yufen Yuan, Jin Zhao, Yong Yang, Yirong Li

**Affiliations:** ^1^ Department of Laboratory Medicine, Zhongnan Hospital of Wuhan University, Wuhan University, Wuhan, Hubei, China; ^2^ Breast Tumor Center, Sun Yat-Sen Memorial Hospital, Sun Yat-Sen University, Guangzhou, Guangdong, China; ^3^ Department of Radiology, Zhongnan Hospital of Wuhan University, Wuhan University, Wuhan, China; ^4^ School of Materials Science and Engineering and School of Electronics and Information technology, Sun Yat-Sen University, Guangzhou, Guangdong, China; ^5^ Department of Pathology, Anyang Tumor Hospital, Anyang, Henan, China; ^6^ Key Laboratory Zoonsis Research Ministry of Education, Institute of Zoonosis, Jilin University, Changchun, Jilin, China

**Keywords:** breast cancer, microarray, ITGA11, Jab1/COPS5, biomarker

## Abstract

Breast cancer is the most prevalent cancer diagnosis in women. We aimed to identify biomarkers for breast cancer prognosis. mRNA expression profiling was performed using Gene Chip Human Transcriptome Array 2.0. Microarray analysis and series test of cluster (STC) analysis were used to screen the differential expressed mRNAs and the expression trend of genes. Immumohistochemical staining with 100 clinical specimens was used to validate two differentially expressed genes, ITGA11 and Jab1. In the present study, significantly enriched Gene Ontology (GO) terms and pathways were identified. 26 model profiles were used to summarize the expression pattern of differentially expressed genes. Results of immunohistochemistry were consistent with those of the microarray, in that ITGA11 and Jab1 were differentially expressed with the same trend. Survival analyses using the Kaplan–Meier method demonstrated that breast cancer patients with high levels of either ITGA11 or Jab1 had a significant association with worse prognosis. Our study identified ITGA11 and Jab1 as novel biomarkers for breast cancer.

## INTRODUCTION

Breast cancer is the most common cancer [1] and also the second leading cause of cancer deaths for women [2]. The lack of better adjuvant therapy remains to be a major challenge in reducing the burden of breast cancer patients. Nowadays, the tumor size, lymph node involvement, and distant metastasis (TNM) staging system of the American Joint Committee on Cancer (AJCC) has been widely recognized, but there is still a lack of worldwide-recognized system or reliable markers predicting the prognosis of breast cancer patients. While applying for neoadjuvant chemotherapy or endocrine therapy, clinicopathological parameters are usually unstable, which complicates the judgment of real prognosis. Therefore, there is a pressing need to find biomarkers for breast cancer which can help to develop better treatment solutions for breast cancer.

Intensive research has been focused on understanding the molecular mechanisms of breast cancer [3]. Many genetic changes that lead to abnormal cellular functions have been identified in breast cancer cells [4, 5]. Multiple factors in the tumor microenvironment further influence the cancer progression via a wide variety of receptors and the corresponding signal pathways [6], which also involve various oncogenes and anti-oncogenes [7, 8]. Microarray data analysis, which features high throughput and high sensitivity, has made it possible to test the expression changes of the whole genome [9]. There have been many reports on gene expression profiling in breast cancer [10, 11]. Therefore, the development of microarray analysis provides new insight in diagnosis and treatment of breast cancer.

Understanding new developments in transcriptome and pathways may identify novel biomarkers for cancer. Integrin α11 (ITGA11), a integrin family members, involve in various processes that influences the cell’s biological behavior, such as metastasis, embryogenesis, hemostasis, immune response, tissue repair, cancer growth, tumor angiogenesis, and resistance to therapy [12, 13]. Alterations in integrin disturb cancer cell adhesion and extracellular matrix assembly, which may further lead to tumor metastasis [14]. Integrins also interact with tyrosine kinase receptors which promote cancer cell proliferation, and differentiation [15].

c-Jun activation domain-binding protein 1 (Jab1), primarily identified as a c-Jun coactivator [16], is the fifth member of the constitutive photomorphogenesis 9 signalosome (COPS5) complex. Jab1/COPS5 regulates cell signal transduction, genetic transcription and protein stability [16]. Importantly, Jab1/COPS5 plays an important role in proliferation and invasiveness [17].

In the current study, we identified potential genes associated with breast cancer tumorigenesis by transcriptional network analysis and further validated ITGA11 from the STC analysis and a differentially expressed gene Jab1/COPS5 in clinical patients.

## RESULTS

### Patient characteristics

In the present study, patients’ median age was 50 years old (range 25–80). 35 cases (43.8%) were menopause. The main histological type was ductal in 74 (92.5%), lobular in 5 (6.25%), medullary in 1 (1.25%). 6 (7.5%) had a grade 1 tumor; 54 (67.5%) had a grade 2 tumor; 20 (25.8%) had a grade 3 tumor. Based on tumor staging system, most patients were defined as stage II (48, 60.0%) and stage I (10, 12.5%). 66 patients (82.5%) had lymph node involvement. 1 patients (1.3%) developed distant metastasis. The patients’ clinicopathologic characteristics were summarized in Table 1.

**Table 1:** Characteristics of breast cancer patients (*n* = 80)

Variable	Number (%)
Age	
<60	66 (82.5%)
≥60	14 (17.5%)
Histological type	
Ductal	74 (92.5%)
Lobular & Medullary	6 (7.5%)
Stage	
I	10 (12.5%)
II	48 (60.0%)
III & IV	22 (27.5%)
Grade	
G1	6 (7.5%)
G2	54 (67.5%)
G3	20 (25.0%)
Tumor size	
T1	9 (11.3%)
T2	48 (60.0%)
T3 & T4	23 (28.7%)
Lymph node metastasis	
No	14 (17.5%)
Yes	66 (82.5%)
Distant metastasis	
No	79 (99.8%)
Yes	1 (1.3%)
HER2	
negative	38 (47.5%)
positive	42 (52.5%)
ER	
negative	40 (50%)
positive	40 (50%)
PR	
negative	46 (57.5%)
positive	34 (42.5%)
Ki-67	
<14%	39 (48.8%)
≥14%	41 (51.2%)
Family history	
No	56 (70.0%)
Yes	24 (30.0%)
Menopause	
No	45 (56.3%)
Yes	35 (43.8%)

### Transcriptome array analysis of mRNA expression in breast cancer

**Figure 1:**
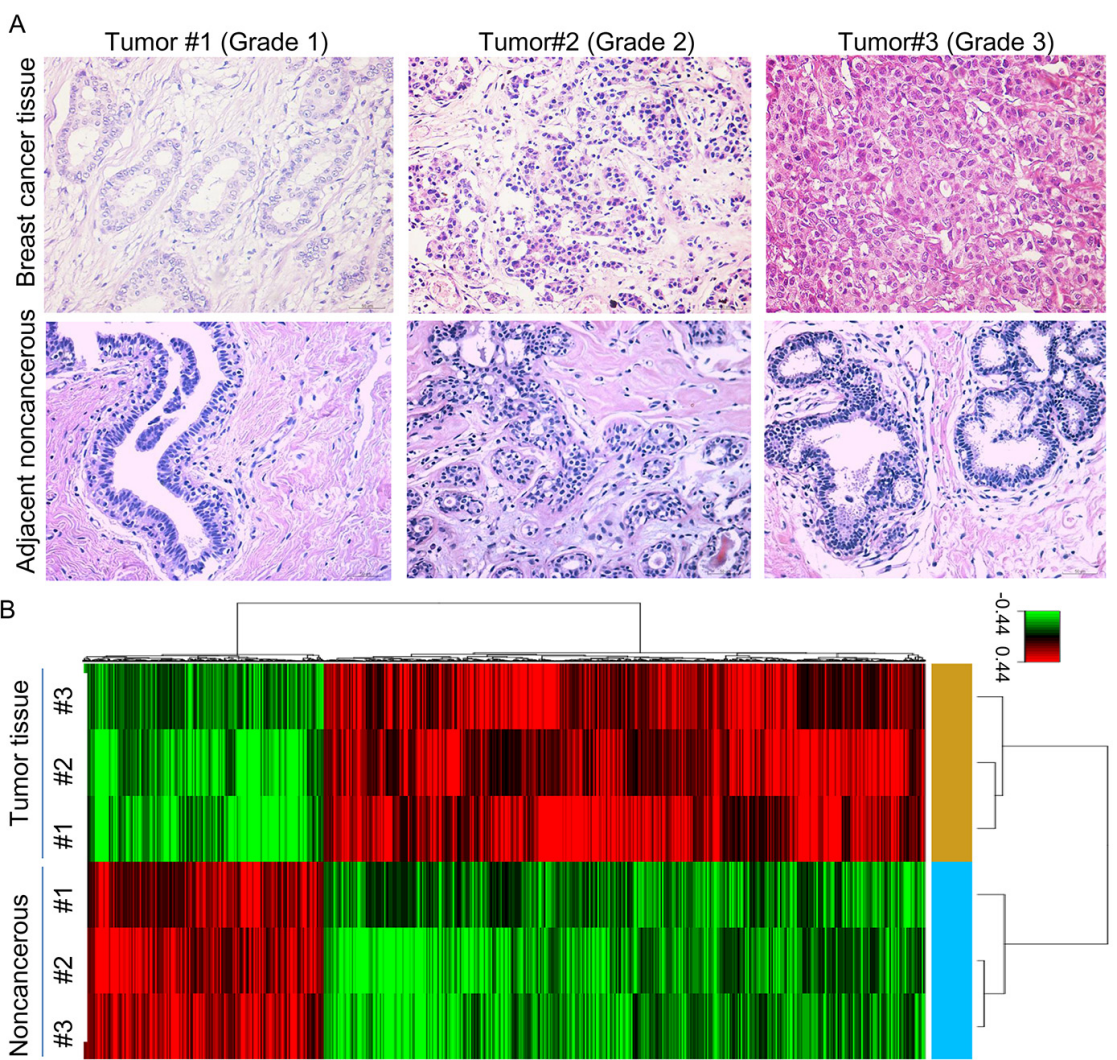
The mRNA profile differentiates breast cancer tissue from adjacent noncancerous breast tissue (**A**) HE staining of breast cancer tissue and adjacent noncancerous breast tissue. (**B**) Hierarchical clustering for the differentially expressed mRNAs (*p* < 0.05).

HE Staining confirmed the breast cancer tissue and paired adjacent noncancerous breast tissue (Figure 1A). We used Affymetrix GeneChip Human Transcriptome Array 2.0 to analyze mRNA expression in the breast tissue, and applied the RVM *t*-test to filter the differentially expressed mRNAs, 509 mRNAs were found to be significantly down-regulated (fold change > 1.2, *p* < 0.05) while 1277 mRNA were markedly up-regulated (fold change > 1.2, *p* < 0.05) in breast cancer tissue compared with the adjacent noncancerous breast tissue (Figure 1B).

**Figure 2:**
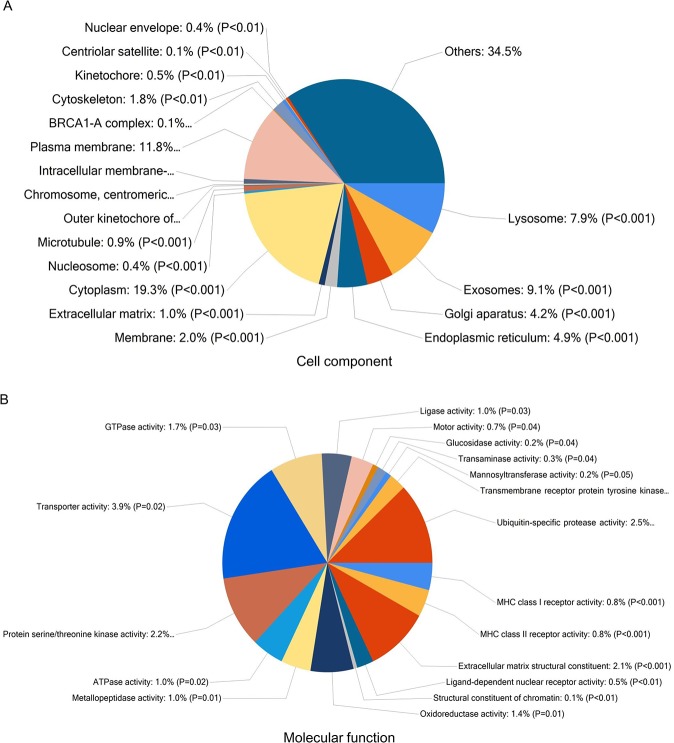
Functional enrichment analysis of genes using FunRich Enrichment of cell component (**A**) and molecular functions (**B**) in dysregulated mRNAs.

**Figure 3:**
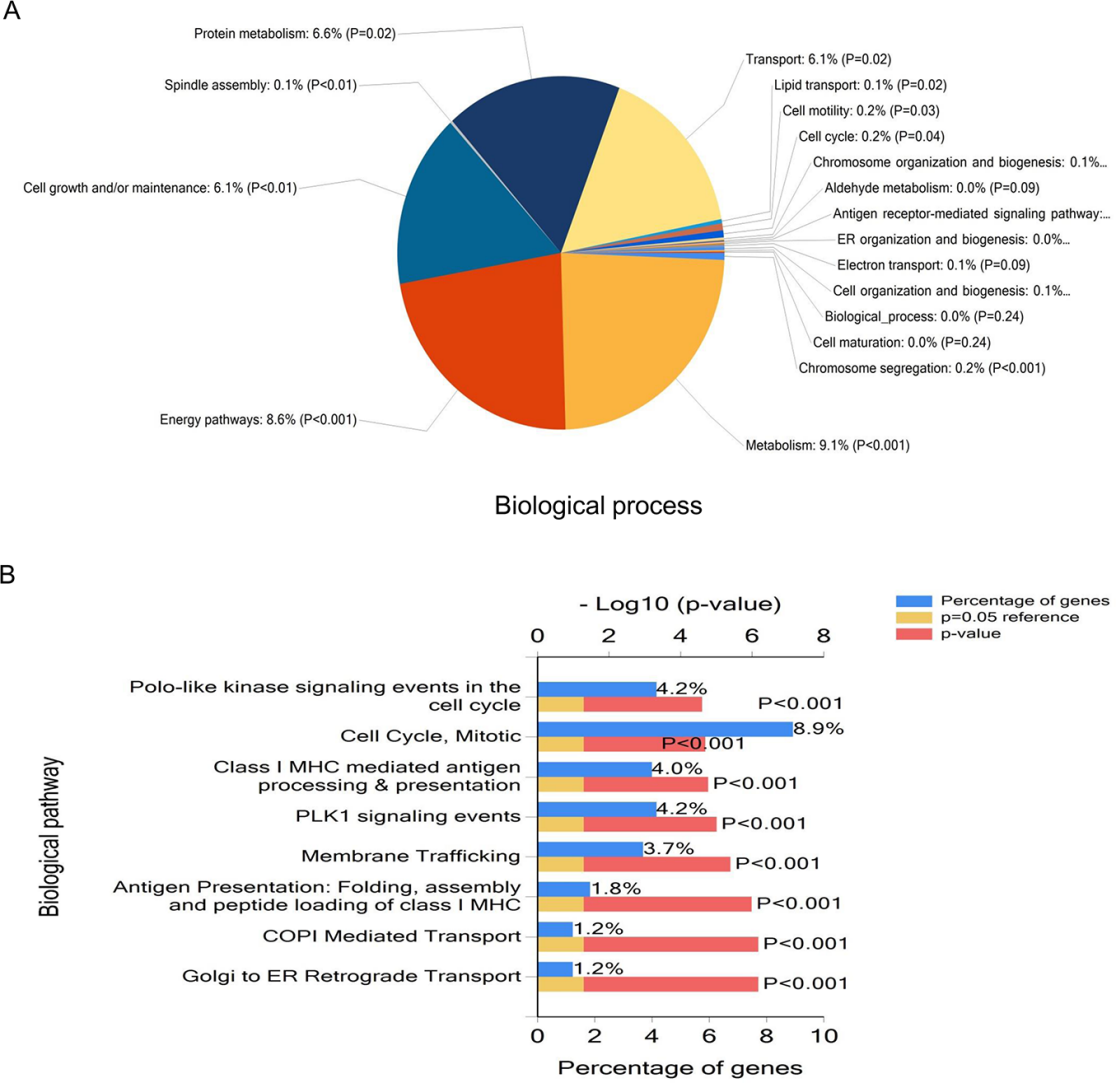
Functional enrichment analysis of genes using FunRich Pie graph of biological processes (**A**) and Bar graph of biological pathways (**B**) in genes are shown.

In order to identify mRNAs that are overrepresented in any functional class, the dysregulated mRNAs were subjected to functional enrichment analysis using FunRich software (Figures 2 and 3). According to the cell component, 19.3% of genes were categorized as cytoplasm, 11.8% of genes were identified in plasma membrane (Figure 2A). Among molecular functions, genes were enriched in transporter activity (3.9%), ubiquitin-specific protease activity (2.5%), protein serine/threonine kinase activity (2.2%) and extracellular matrix structural constituent (2.1%) (Figure 2B).

Further to this, genes involved in metabolism, energy pathways, cell growth and/or maintenance and protein metabolism were enriched in (Figure 3A). In the context of biological pathway, cell cycle, mitotic, polo-like kinase signaling events in the cell cycle, PLK1 signaling events membrane trafficking were significantly overrepresented in mRNAs (Figure 3B).

### STC analysis

**Figure 4:**
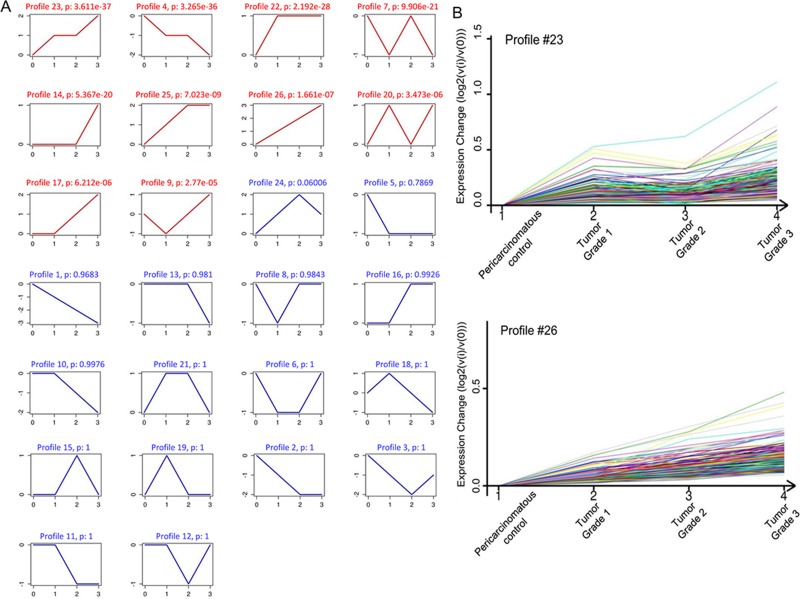
STC analysis for differential genes related to tumor grade (**A**) The expression patterns of genes were analyzed and 26 model profiles were used to summarize. Each box represents a model expression profile. Each image corresponds to a different model grade expression profile. The upper number in the profile box is the model profile number and the *P* value. The horizontal axis represents group points, and the vertical axis shows the gene expression levels for the gene after Log normalized transformation. (**B**) Two expression patterns of genes showed significant *P* values (*P* < 0.001). The number of genes assigned to each model profile is used as the estimate of the number of co-expressed genes.

To further narrow the range of target genes with high significance, the tumor grade-serial expression pattern of significantly gene was investigated. Each profile consists of a cluster of multiple genes that have similar expression patterns with increasing tumor grade. As shown in Figure 4A, 26 model profiles were used to summarize the expression pattern of these genes. Each box represents a model profile. Among the 26 patterns, totally 10 expression patterns including profiles 23, 4, 22, 7, 14, 25, 26, 20, 17 and 9 showed significant *P* values (*P* < 0.001). Six of these clusters contained genes which were stable (profile 22 and 14) or gradually elevated (profiles 23, 25, 26 and 17), while genes in profile 4 had opposite effects to profile 23. Profile 9 contained genes which were suppressed at grade 1 points and then gradually increased expression levels at higher tumor grades points (Figure 4B).

### ITGA11 and Jab1 overexpressed in breast cancer patients

**Figure 5:**
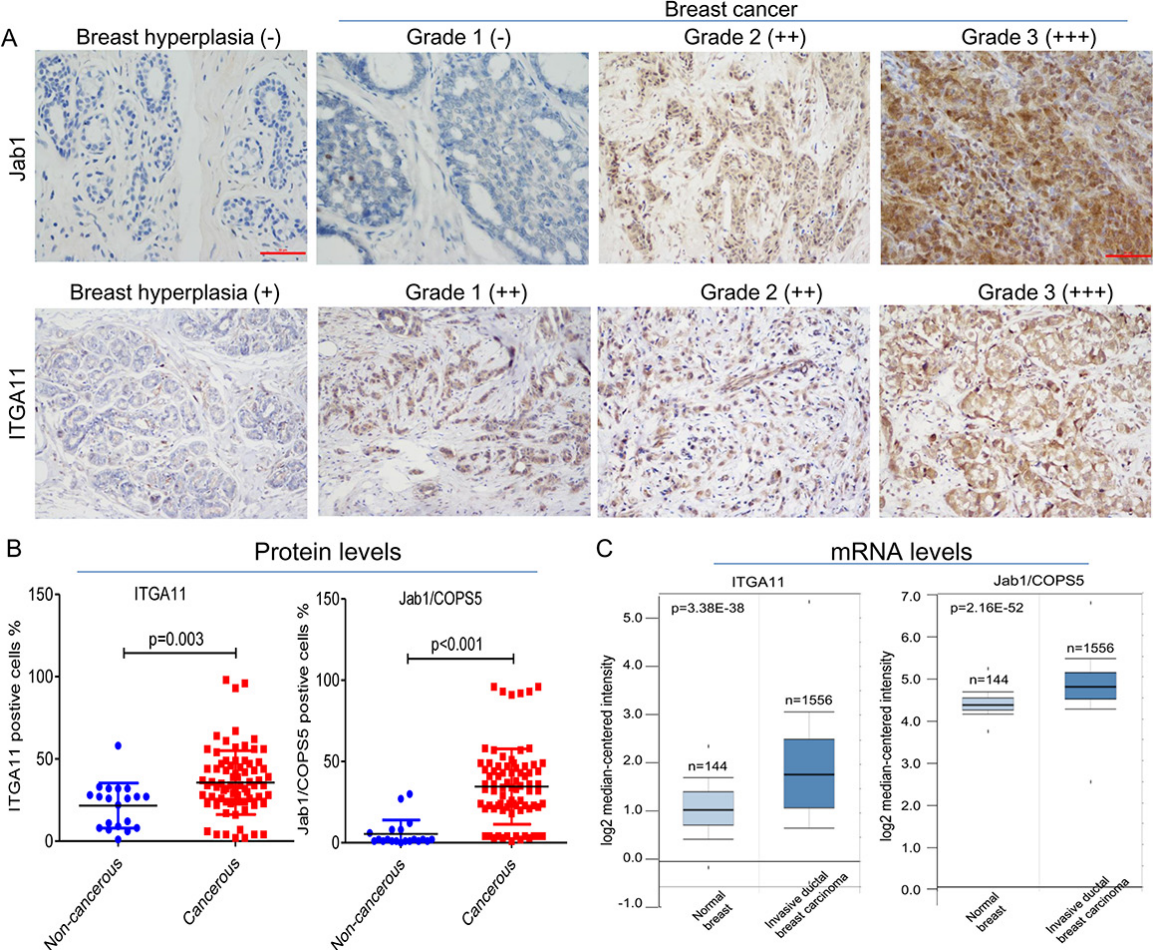
IGTA11 and Jab1 expressed in breast cancer (**A**) Immunohistochemical analysis of IGTA11 and Jab1 in breast hyperplasia and breast cancer tissue. Original magnification, ×200. (**B**) The percentages of postive IGTA11 or positive Jab1 cell in noncancerous and cancerous tissues. (**C**) *IGTA11* and *Jab1* gene expression in normal breast and breast cancer using the Oncomine gene expression tool (https://www.oncomine.com). The clinical data were downloaded from Oncomine Data Portal.

To validate the findings from our transcriptome array analysis, immunohistochemical staining of ITGA11 and Jab1/Cops5 was conducted in 80 breast cancer tissue and 20 noncancerous breast tissue. As expected, both ITGA11 and Jab1/Cops5 were overexpressed in breast cancer (Figure 5A and 5B). In agreement with the immunohistochemistry findings, the data from online database ONCOMINE indicated that ITGA11 and Jab1/Cops5 mRNA expression levels in breast cancer are much higher than those in normal breast tissue (Figure 5C).

### Association between ITGA11 and Jab1 and clinicopathological parameters

Correlations between ITGA11 and Jab1/Cops5 and clinicopathological parameters were summarized in Table 2. Elevated ITGA11 levels were associated with higher tumor grade (G1 *vs*. G2 and G3, 20.0% *vs*. 34.9% and 42.1%, respectively, *p* = 0.044). Jab1 levels were not only associated with tumor grade (G1 *vs*. G2 and G3, 26.7% *vs*. 31.4% and 45.7%, respectively, *p* = 0.040), but also associated with ER status (negative *vs*. positive, 27.5% *vs*. 41.7%, *p* = 0.005) and PR status (negative vs. positive, 30.0% vs. 40.8%, *p* = 0.039). However, we did not observe any association between ITGA11/ Jab1 and histological type, tumor stage, family history nor menopause.

**Table 2:** Correlation between ITGA11/Jab1 and clinicopathological parameters

Variable	ITGA11 postive cells %	*P* value	Jab1 postive cells %	*P* value
Age				
<60	35.4 ± 20.2	0.854	34.5 ± 22.2	0.972
≥60	36.5 ± 15.4		34.8 ± 28.4	
Histological type				
Ductal	36.3 ± 19.4	0.310	34.5 ± 23.6	0.921
Lobular & Medullary	27.8 ± 19.4		35.5 ± 19.4	
Stage				
I	25.9 ± 13.2	0.210	26.1 ± 17.7	0.104
II	37.8 ± 17.8		39.0 ± 25.6	
III & IV	35.2 ± 24.9		28.7 ± 17.7	
Grade				
G1	20.0 ± 10.5	0.044	26.7 ± 22.4	0.040
G2	34.9 ± 16.0		31.4 ± 15.0	
G3	42.1 ± 26.4		45.7 ± 35.9	
Tumor size				
T1	26.7 ± 10.8	0.314	26.1 ± 15.5	0.100
T2	37.4 ± 18.4		39.1 ± 25.9	
T3 & T4	35.3 ± 23.4		28.5 ± 17.3	
LN metastasis				
No	30.0 ± 15.7	0.235	35.1 ± 24.4	0.922
Yes	36.8 ± 20.0		34.5 ± 23.1	
HER2				
negative	36.8 ± 22.7	0.597	36.9 ± 22.6	0.395
positive	34.5 ± 16.1		32.5 ± 23.8	
ER				
negative	33.2 ± 18.3	0.262	27.5 ± 20.0	0.005
positive	38.1 ± 20.3		41.7 ± 24.2	
PR				
negative	34.8 ± 16.5	0.646	30.0 ± 22.1	0.039
positive	36.8 ± 22.9		40.8 ± 23.5	
Ki-67				
<14%	36.3 ± 17.3	0.752	34.5 ± 25.8	0.985
≥14%	34.9 ± 21.4		34.6 ± 20.7	
Family history				
No	37.8 ± 21.3	0.123	37.4 ± 23.2	0.092
Yes	30.5 ± 13.1		27.9 ± 22.2	
Menopause				
No	35.1 ± 20.5	0.773	36.6 ± 23.7	0.387
Yes	36.3 ± 18.2		32.0 ± 22.6	

Abbreviations: LN: lymph node.

**Figure 6:**
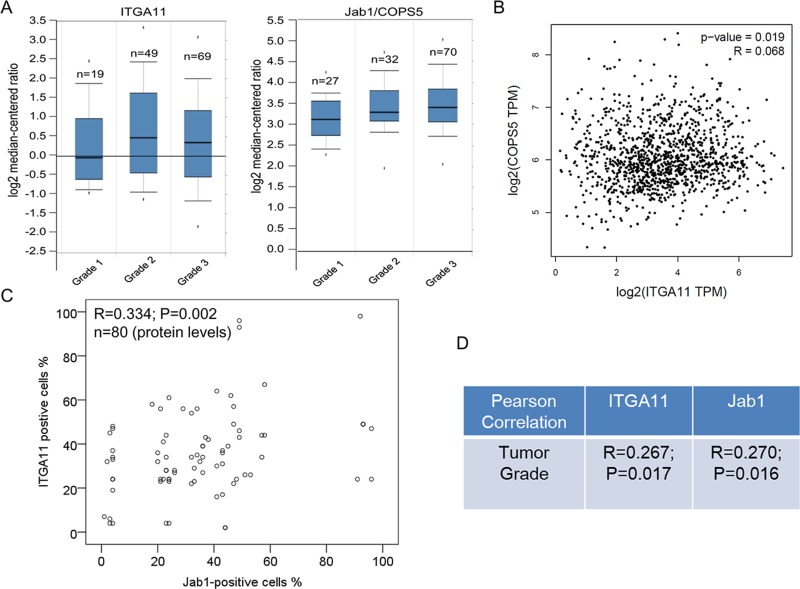
IGTA11 and Jab1 associates with tumor grade in breast cancer (**A**) *IGTA11* and *Jab1* gene expression in breast cancer with different tumor grade using the Oncomine gene expression tool. (**B**) Overview of the two genes in breast cancer across online data GEPIA. (**C**) IGTA11 was associated with Jab1 expression in breast cancer from the immunohistochemistry data. (**D**) IGTA11 and Jab1 associated with tumor grade in breast cancer from the immunohistochemistry data. The *R* and *P* values were from Pearson Correlation.

In agreement with the immunohistochemistry results, data from ONCOMINE indicated that ITGA11 and Jab1/Cops5 expression levels tend to be higher in breast cancer with higher grade (Figure 6A). Furthermore, RNA sequencing analysis of mRNA expression from the GEPIA online database revealed that ITGA11 was associated with Jab1/Cops5 in breast cancer patients (Figure 6B). In addition, our immunohistochemistry data in breast cancer tissue indicated that Jab1 level was associated with ITGA11 levels in breast cancer (Figure 6C) and both ITGA11 and Jab1 levels were correlated with tumor grade (Figure 6D).

### Survival analysis

**Figure 7:**
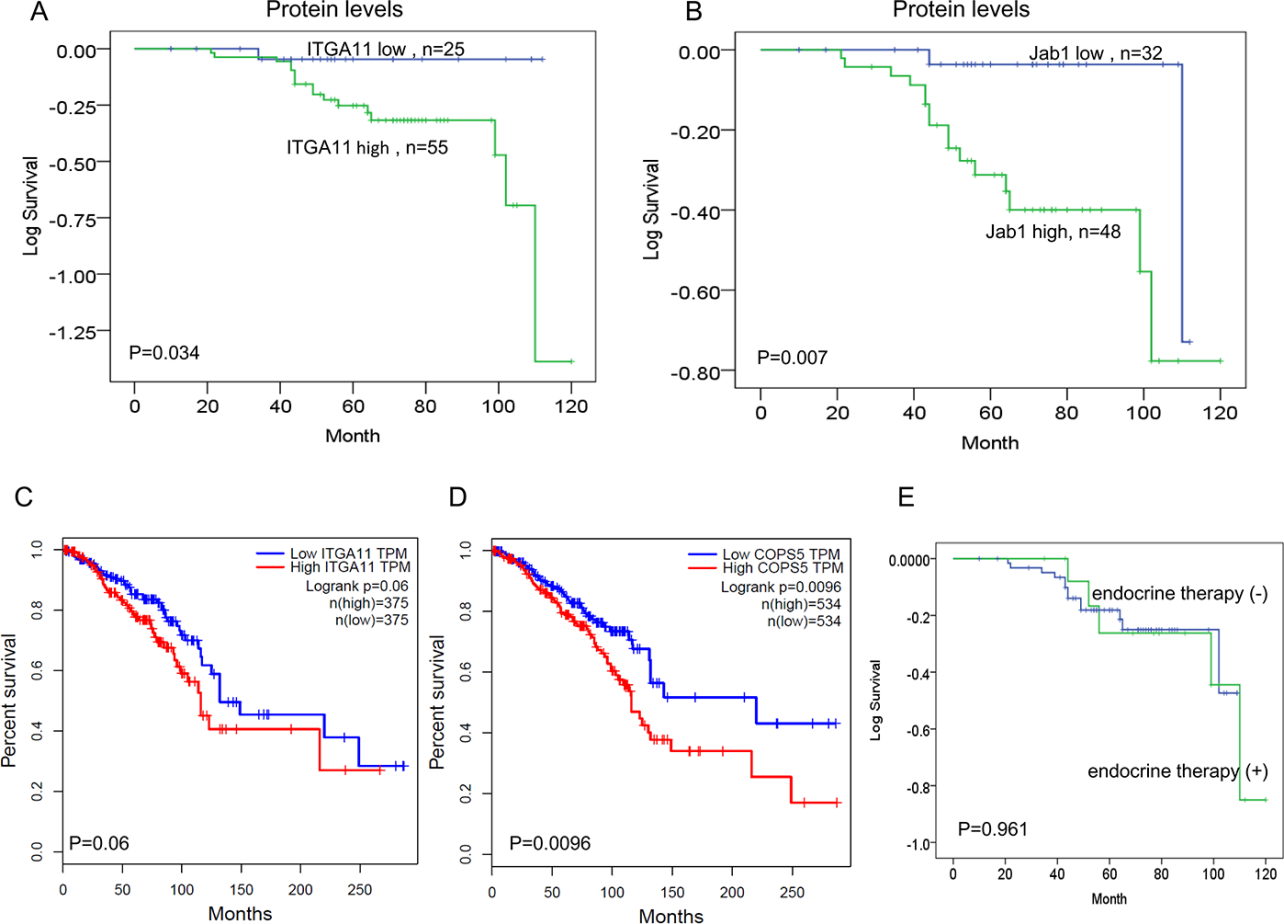
IGTA11 and Jab1/COPS5 predict survival in breast cancer (**A**, **B**) Kaplan–Meier analyses of the association between IGTA11 and Jab1 protein expression and survival. (**C**, **D**) *In vivo* RNA sequencing data and Kaplan–Meier plots from GEPIA database were used to assess correlations between *IGTA11* or Jab1/COPS5 gene expression and breast cancer patients survival. (**E**) Kaplan–Meier analyses of the association between endocrine therapy and survival.

The median follow-up time was 64 months (range 10–120). In order to evaluate the prognostic influence of ITGA11 and Jab1/COPS5 expression, we carried out Kaplan–Meier analyses to compare grouped patients. The survival curves demonstrated that patients with high levels of ITGA11 or Jab1/COPS5 had a significant association with worse OS (*p* = 0.034, *p* = 0.007; Figure 7A and 7B).

We also investigated the relationship between ITGA11 and Jab1/COPS5 and survival of breast cancer patients in GEPIA database. The online data was consistent with the IHC data, suggesting high levels of either ITGA11 or Jab1/COPS5 was associated with worse survival in breast cancer (Figure 7C and 7D). However, we didn't find significant influence of endocrine therapy on the survival (Figure 7E). We also analysed the survival in different subtypes of breast cancer and found similar results (Supplementary Figures 1 and 2).

## DISCUSSION

Breast cancer is the most frequent cancer among women worldwide. Screening and diagnosis of breast cancer at earlier stages are of great importance to improve patient survival and reduce treatment costs. However, the underlying mechanism regulating breast cancer aggressiveness remain poorly understood, and biomarkers for the detection of early-stage breast cancer are still lacking.

The advent of high throughput mRNA microarray analysis method makes it possible to detect the expression of thousands of mRNAs, which allows us to have a clearer picture of the global transcriptome in both cancer tissue and normal tissue [18]. In this study, we assessed the mRNA expression profiles in both breast cancer tissue and paired noncancerous breast tissue using microarray technique and explored their possible functions using GO analysis, KEGG pathway analysis and STC analysis. A number of mRNAs were significantly differentially expressed in breast cancer tissue compared with noncancerous breast tissue. In order to validate the microarray results, we further carried out independent measurement of ITGA11 and Jab1 protein levels in breast cancer and noncancerous tissue samples using immunohistochemistry. The immunohistochemistry results showed good consistency with microarray.

We also performed GO analysis, KEGG pathway analysis, and STC analysis to identify the enriched biological functions among the differentially expressed genes. It was found that the genes were participated in a variety of molecular functions, cellular components, and biological processes. Many pathways related to cancer have been identified by the pathway analysis, among which “cell cycle” and “PLK1 signaling events” are two of the most enriched pathways. Cell cycle is a highly organized and regulated process that ensures duplication of genetic material and cell division. Proliferation depends on progression through four distinct phases of the cell cycle, which is regulated by several cyclin-dependent kinases (CDKs) and their cyclin partners [19]. Cancer growth is caused by abrogation of appropriate cell-cycle control, and many cell-cycle kinases are amplified or overexpressed in cancer [19]. Polo-like kinase 1 (PLK1) plays a vital role in cell cycle progression through mitosis via its effects on chromosome segregation, spindle assembly and cytokinesis [20]. Inhibition of PLK1 delay acentriolar spindle formation during mitosis and promote apoptosis [21]. Further, PLK1 is an important regulator of the DNA damage checkpoint [22]. PLK1 is overexpressed in a variety of malignancy including breast cancer [23, 24]. Additionally, PLK1 overexpression is associated with poor prognosis in cancer patients [25]. These results demonstrated the reliability of our microarray study.

Integrins are heterodimeric cell surface adhesion receptors contains α and β subunits. Twenty-four distinct integrin heterodimers are expressed in mammals as a result of combinatorial association of 18 α and 8 β subunits [26]. Extracellular matrix (ECM) ligands can bind to the α subunit and activate intracellular signaling events via the β subunit to integrate extracellular and intracellular events necessary for cell motility and invasion [14]. Many integrins are expressed at low or undetectable levels in adult epithelia, but are up-regulated in tumors [26]. Integrin α11 (ITGA11) is expressed in many tissues in the embryo but disappears with maturation in adult tissues [27]. However, it has been proved that its expression is up-regulated in malignant conditions such as non-small-cell lung carcinoma, where it has been suggested to be connected to cancer cell growth [28, 29].

We found high expression of ITGA11 was correlated with poor prognosis in breast cancer patients, which is in agreement with previous studies that Integrin expression levels are correlated with prognosis in glioblastoma, melanoma, gastric cancer, cervical cancer, and ovarian cancer [30–33].

Aberrant overexpression of Jab1/COPS5 is demonstrated to play a role in the pathogenesis of several types of human cancers and correlate with poor cancer prognosis [34, 35]. Jab1/COPS5 isopeptidase activity is essential for human and murine mammary epithelial transformation and progression [36]. Jab1/COPS5 expression was low in or absent from normal breast tissue, while it was abnormally expressed in breast tumors [37]. Importantly, breast cancer patients with Jab1/COPS5-negative tumors had neither relapse nor disease progression at a median follow-up time of 70 months [38]. In line with these results, our study found that higher levels of Jab1 expression in breast cancer patients compared with that in non-cancerous tissue and Jab1 expression was associated with tumor grade, suggesting an role of Jab1 in tumor progress.

Taken together, we have identified ITGA11 and Jab1 as biomarkers in breast cancer. High throughput microarray data analysis may act as an efficient tool to discover more prognostic markers and therapeutic targets in breast cancer.

## MATERIALS AND METHODS

### Patients and tissue samples

Three breast cancer tissue and three paired adjacent noncancerous breast tissue specimens were from Anyang Tumor Hospital (Anyang, Henan, China). Other 80 cases of breast cancer patients and 20 cases of breast hyperplasia patients treated at Anyang Tumor Hospital from July 2007 to July 2012 were randomly included in this study for immunohistochemical analysis. Breast hyperplasia is a diagnostic category of proliferative disease that includes inflammatory hyperplasia, atypical ductal hyperplasia and atypical lobular hyperplasia. The inclusion criteria for the participants were: aged 18 years above; diagnosis of breast cancer. Exclusion criteria were: preoperative chemotherapy or radiotherapy; deficiency of clinical data or lack of follow up. The diagnosis of breast cancer and hyperplasia was confirmed pathologically. Patients who had preoperative diagnosis and had not received preoperative chemoradiotherapy were selected in our study based on the availability of archived paraffin-embedded tissue blocks for immunohistochemistry. Ethical approval from Anyang Tumor Hospital and informed consent from patients have been obtained. The clinical and pathological characteristics of 80 breast cancer patients were summarized in Table 1.

### RNA isolation and transcriptome array

Total RNA in the samples was extracted using Trizol reagent. The integrity and concentration of all RNA samples were measured using the NanoDrop 1000 spectrophotometer. The total RNA extracted from three breast cancer tissue and three paired adjacent noncancerous breast tissue specimens were hybridized to an Affymetrix GeneChip Human Transcriptome Array 2.0. The arrays were scanned by GeneChip^®^ Command Console^®^ Software and the acquired array images were analyzed by Affymetrix GeneChip Operating Software.

### Cluster analysis and series test of cluster (STC) analysis

Differential expressed genes from microarray data were screened by applying random variance model (RVM) *t*-test and considered to be down or up regulated with *p* < 0.05. The cluster analysis of genes was accordingly conducted through GCBI online system (https://www.gcbi.com.cn/gcuser/html/member/home). STC algorithm of gene expression was performed to profile the gene expression with grade malignancy series and to identify the most probable set of clusters generating grade malignancy series. Dynamic nature of gene expression profiles was taken into account in STC and therefore it can identify the number of distinct clusters. Fisher’s exact test was used to examine significant profiles, and *p* < 0.05 was considered as the threshold of significance.

### Functional enrichment analysis

mRNAs identified in breast cancer were subjected to Gene Ontology (GO) and biological pathway enrichment analysis using FunRich tool (http://www.funrich.org) against human FunRich background database.

### Analysis of clinical mRNA microarrays for the detection of correlations between ITGA11 and patients survival

Transcriptome data from patient samples of breast cancer were analyzed using the online database ONCOMINE (https://www.oncomine.org/resource/login.html) to investigate whether the expression of the markers are associated with tumor grade. RNA sequencing analysis and visualization platform GEPIA (http://gepia.cancer-pku.cn/) was used to determine whether the expression levels of ITGA11 and Jab1 were correlated in breast cancer. GEPIAwas used to determine whether the expression of ITGA11 and Jab1 was correlated with the breast cancer patients’ overall survival.

### Immunohistochemical analysis

The tissues were processed routinely and stained with hematoxylin and eosin (HE) [39]. ITGA11 and Jab1 levels in the formalin-fixed, paraffin-embedded tissue were evaluated using immunohistochemical staining, as described in our previous work [40]. Briefly, the samples were sectioned and mounted on slides, following drying at 60°C for 1 hour. Slides were then deparaffinized in 2-xylene. To retrieval antigen, the slides were boiled for 3 minutes in 0.01 mol/L sodium citrate (pH 6.0) and then cooled at room temperature for 30 minutes. To block the Endogenous peroxidase activity, the slides were further immersed in 0.3% H_2_O_2_. Then the slides were incubated with the primary antibodies ITGA11 (Santa Cruz, sc-98740) and Jab1 (Santa Cruz, sc-13157) diluted at 1:200 overnight at 4°C and were detected by a secondary antibody kit (Dako Corp). ITGA11 and Jab1 expression were measured by counting no less than 400 tumor cells. Tumor cells were considered positive for markers when nuclear or cytoplasmic staining was present. The positivity represented the estimated fraction of positively stained cells (−, ≤5%; +, 5% to 25%; ++, 26% to 50%; +++, >50%). All experiments were performed in accordance with approved guidelines and regulations of Anyang Tumor Hospital.

### Follow-up and statistical analysis

Overall survival (OS) was calculated as the period from initial diagnosis to death regardless of breast cancer related or not. Before closing the research database, the authors updated the follow-up data of patients who had not visited our outpatient department for more than three months. Patient follow-up was censored at the time of death or finalization of the study. Percent of ITGA11 positive cells and Jab1 positive cells were presented as means ± standard deviation (SD). Categorical variables were presented as numbers and percentages. Comparisons between groups were carried out with the *T* test or the one-way ANOVA and LSD tests for continuous variables. Multivariate survival analyses were performed to identify independent factors for overall survival. Kaplan–Meier method was applied for performing stratified overall survival analysis, followed by the log-rank test. It was regarded as statistically significant when *P* < 0.05. Calculations were performed using IBM SPSS statistics software 22.0.

## SUPPLEMENTARY FIGURES



## Abbreviations

STC: series test of cluster
GO: Gene Ontology
HE: hematoxylin and eosin
OS: Overall survival
ECM: Extracellular matrix
CDK: cyclin-dependent kinase
PLK1: Polo-like kinase 1

## References

[1] Torre LA, Bray F, Siegel RL, Ferlay J, Lortet-Tieulent J, Jemal A (2015). Global cancer statistics, 2012. CA Cancer J Clin.

[2] DeSantis C, Ma J, Bryan L, Jemal A (2014). Breast cancer statistics, 2013. CA Cancer J Clin.

[3] Li Z, Dong M, Fan D, Hou P, Li H, Liu L, Lin C, Liu J, Su L, Wu L, Li X, Huang B, Lu J (2017). LncRNA ANCR down-regulation promotes TGF-beta-induced EMT and metastasis in breast cancer. Oncotarget.

[4] Takeshita T, Yamamoto Y, Yamamoto-Ibusuki M, Tomiguchi M, Sueta A, Murakami K, Omoto Y, Iwase H (2017). Analysis of ESR1 and PIK3CA mutations in plasma cell-free DNA from ER-positive breast cancer patients. Oncotarget.

[5] Ma F, Li W, Liu C, Li W, Yu H, Lei B, Ren Y, Li Z, Pang D, Qian C (2017). MiR-23a promotes TGF-beta1-induced EMT and tumor metastasis in breast cancer cells by directly targeting CDH1 and activating Wnt/beta-catenin signaling. Oncotarget.

[6] Desmedt C, Yates L, Kulka J (2016). Catalog of genetic progression of human cancers: breast cancer. Cancer Metastasis Rev.

[7] Guerrero-Zotano A, Mayer IA, Arteaga CL (2016). PI3K/AKT/mTOR: role in breast cancer progression, drug resistance, and treatment. Cancer Metastasis Rev.

[8] Bayraktar S, Arun B (2017). BRCA mutation genetic testing implications in the United States. Breast.

[9] Zhu T, Gao YF, Chen YX, Wang ZB, Yin JY, Mao XY, Li X, Zhang W, Zhou HH, Liu ZQ (2017). Genome-scale analysis identifies GJB2 and ERO1LB as prognosis markers in patients with pancreatic cancer. Oncotarget.

[10] Makoukji J, Makhoul NJ, Khalil M, El-Sitt S, Aldin ES, Jabbour M, Boulos F, Gadaleta E, Sangaralingam A, Chelala C, Boustany RM, Tfayli A (2016). Gene expression profiling of breast cancer in Lebanese women. Sci Rep.

[11] Wang J, Yang X, Chen H, Wang X, Wang X, Fang Y, Jia Z, Gao J (2017). A high-throughput method to detect RNA profiling by integration of RT-MLPA with next generation sequencing technology. Oncotarget.

[12] Schwartz MA, Ginsberg MH (2002). Networks and crosstalk: integrin signalling spreads. Nat Cell Biol.

[13] Nam K, Son SH, Oh S, Jeon D, Kim H, Noh DY, Kim S, Shin I (2017). Binding of galectin-1 to integrin beta1 potentiates drug resistance by promoting survivin expression in breast cancer cells. Oncotarget.

[14] Hynes RO (2002). Integrins: bidirectional, allosteric signaling machines. Cell.

[15] Hehlgans S, Haase M, Cordes N (2007). Signalling via integrins: implications for cell survival and anticancer strategies. Biochim Biophys Acta.

[16] Claret FX, Hibi M, Dhut S, Toda T, Karin M (1996). A new group of conserved coactivators that increase the specificity of AP-1 transcription factors. Nature.

[17] Adler AS, Lin M, Horlings H, Nuyten DS, van de Vijver MJ, Chang HY (2006). Genetic regulators of large-scale transcriptional signatures in cancer. Nat Genet.

[18] Kadara H, Fujimoto J, Yoo SY, Maki Y, Gower AC, Kabbout M, Garcia MM, Chow CW, Chu Z, Mendoza G, Shen L, Kalhor N, Hong WK (2014). Transcriptomic architecture of the adjacent airway field cancerization in non-small cell lung cancer. J Natl Cancer Inst.

[19] Otto T, Sicinski P (2017). Cell cycle proteins as promising targets in cancer therapy. Nat Rev Cancer.

[20] de Carcer G, Manning G, Malumbres M (2011). From Plk1 to Plk5: functional evolution of polo-like kinases. Cell Cycle.

[21] Liu X, Erikson RL (2003). Polo-like kinase (Plk)1 depletion induces apoptosis in cancer cells. Proc Natl Acad Sci USA.

[22] Takaki T, Trenz K, Costanzo V, Petronczki M (2008). Polo-like kinase 1 reaches beyond mitosis--cytokinesis, DNA damage response, and development. Curr Opin Cell Biol.

[23] Takai N, Hamanaka R, Yoshimatsu J, Miyakawa I (2005). Polo-like kinases (Plks) and cancer. Oncogene.

[24] Iyer RS, Nicol SM, Quinlan PR, Thompson AM, Meek DW, Fuller-Pace FV (2014). The RNA helicase/transcriptional co-regulator, p68 (DDX5), stimulates expression of oncogenic protein kinase, Polo-like kinase-1 (PLK1), and is associated with elevated PLK1 levels in human breast cancers. Cell Cycle.

[25] Tokumitsu Y, Mori M, Tanaka S, Akazawa K, Nakano S, Niho Y (1999). Prognostic significance of polo-like kinase expression in esophageal carcinoma. Int J Oncol.

[26] Desgrosellier JS, Cheresh DA (2010). Integrins in cancer: biological implications and therapeutic opportunities. Nat Rev Cancer.

[27] Popova SN, Lundgren-Akerlund E, Wiig H, Gullberg D (2007). Physiology and pathology of collagen receptors. Acta Physiol (Oxf).

[28] Zhu CQ, Popova SN, Brown ER, Barsyte-Lovejoy D, Navab R, Shih W, Li M, Lu M, Jurisica I, Penn LZ, Gullberg D, Tsao MS (2007). Integrin alpha 11 regulates IGF2 expression in fibroblasts to enhance tumorigenicity of human non-small-cell lung cancer cells. Proc Natl Acad Sci USA.

[29] Navab R, Strumpf D, To C, Pasko E, Kim KS, Park CJ, Hai J, Liu J, Jonkman J, Barczyk M, Bandarchi B, Wang YH, Venkat K (2016). Integrin α11β1 regulates cancer stromal stiffness and promotes tumorigenicity and metastasis in non-small cell lung cancer. Oncogene.

[30] Zhang ZY, Xu KS, Wang JS, Yang GY, Wang W, Wang JY, Niu WB, Liu EY, Mi YT, Niu J (2008). Integrin alphanvbeta6 acts as a prognostic indicator in gastric carcinoma. Clin Oncol (R Coll Radiol).

[31] Elayadi AN, Samli KN, Prudkin L, Liu YH, Bian A, Xie XJ, Wistuba II, Roth JA, McGuire MJ, Brown KC (2007). A peptide selected by biopanning identifies the integrin alphavbeta6 as a prognostic biomarker for nonsmall cell lung cancer. Cancer Res.

[32] Goldberg I, Davidson B, Reich R, Gotlieb WH, Ben-Baruch G, Bryne M, Berner A, Nesland JM, Kopolovic J (2001). Alphav integrin expression is a novel marker of poor prognosis in advanced-stage ovarian carcinoma. Clin Cancer Res.

[33] Schittenhelm J, Schwab EI, Sperveslage J, Tatagiba M, Meyermann R, Fend F, Goodman SL, Sipos B (2013). Longitudinal expression analysis of alphav integrins in human gliomas reveals upregulation of integrin alphavbeta3 as a negative prognostic factor. J Neuropathol Exp Neurol.

[34] Pan Y, Yang H, Claret FX (2014). Emerging roles of Jab1/CSN5 in DNA damage response, DNA repair, and cancer. Cancer Biol Ther.

[35] Pan Y, Claret FX (2012). Targeting Jab1/CSN5 in nasopharyngeal carcinoma. Cancer Lett.

[36] Adler AS, Littlepage LE, Lin M, Kawahara TL, Wong DJ, Werb Z, Chang HY (2008). CSN5 isopeptidase activity links COP9 signalosome activation to breast cancer progression. Cancer Res.

[37] Esteva FJ, Sahin AA, Rassidakis GZ, Yuan LX, Smith TL, Yang Y, Gilcrease MZ, Cristofanilli M, Nahta R, Pusztai L, Claret FX (2003). Jun activation domain binding protein 1 expression is associated with low p27(Kip1) levels in node-negative breast cancer. Clin Cancer Res.

[38] Kouvaraki MA, Rassidakis GZ, Tian L, Kumar R, Kittas C, Claret FX (2003). Jun activation domain-binding protein 1 expression in breast cancer inversely correlates with the cell cycle inhibitor p27(Kip1). Cancer Res.

[39] Pan Y, Yuan Y, Liu G, Wei Y (2017). P53 and Ki-67 as prognostic markers in triple-negative breast cancer patients. PLoS One.

[40] Pan Y, Wang S, Su B, Zhou F, Zhang R, Xu T, Zhang R, Leventaki V, Drakos E, Liu W, Claret FX (2017). Stat3 contributes to cancer progression by regulating Jab1/Csn5 expression. Oncogene.

