# Supplementary material for: Analysis of differential gene expression profile identifies novel biomarkers for breast cancer
Source: Oncotarget. 2017 Dec 8;8(70):114613–25. doi: 10.18632/oncotarget.23061 (PMC5777718; doi:10.18632/oncotarget.23061)
Supplement: Supplementary file 1 [file oncotarget-08-114613-s001.pdf]

Analysis of differential gene expression profile identifies novel biomarkers for breast cancer

SUPPLEMENTARY MATERIALS

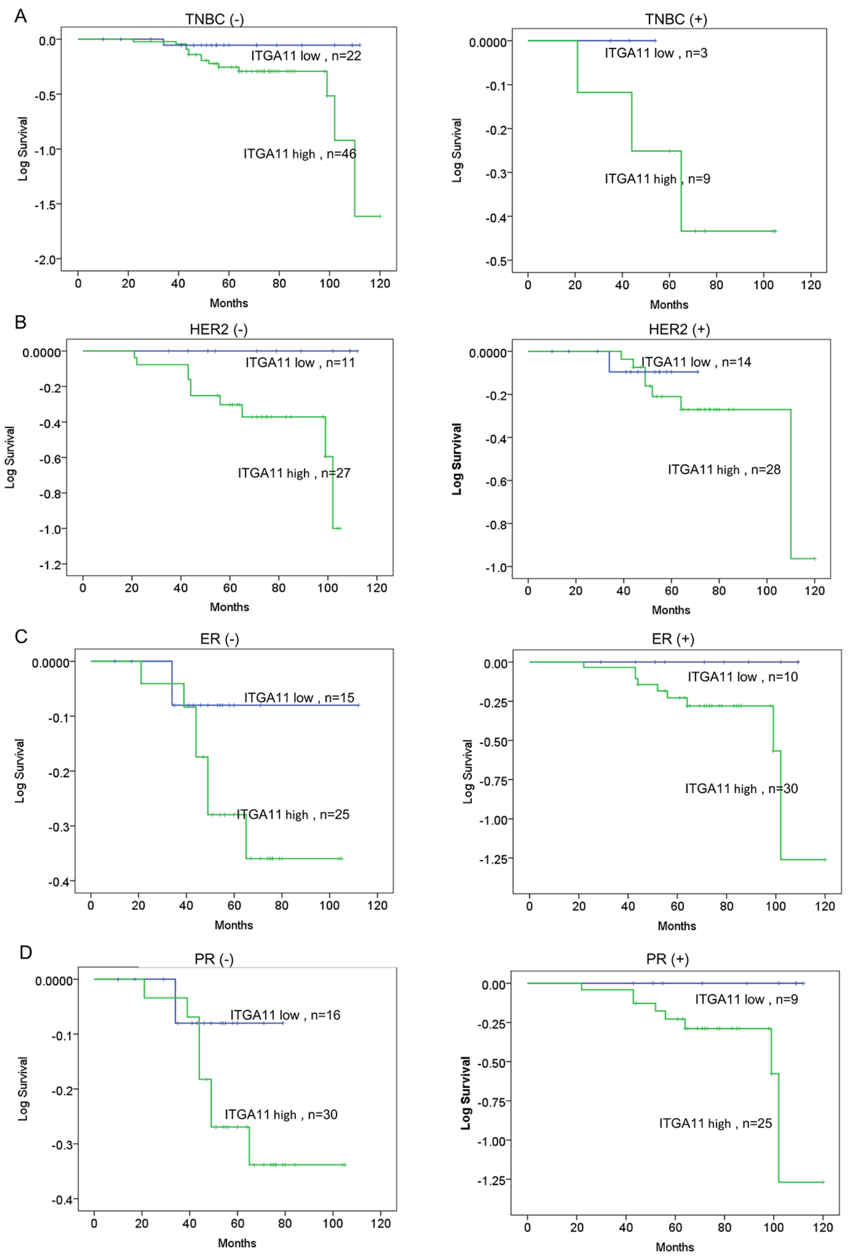

Supplementary Figure 1: Kaplan-Meier analyses of the association between IGTA11 protein expression and survival in different subtype of breast cancer.

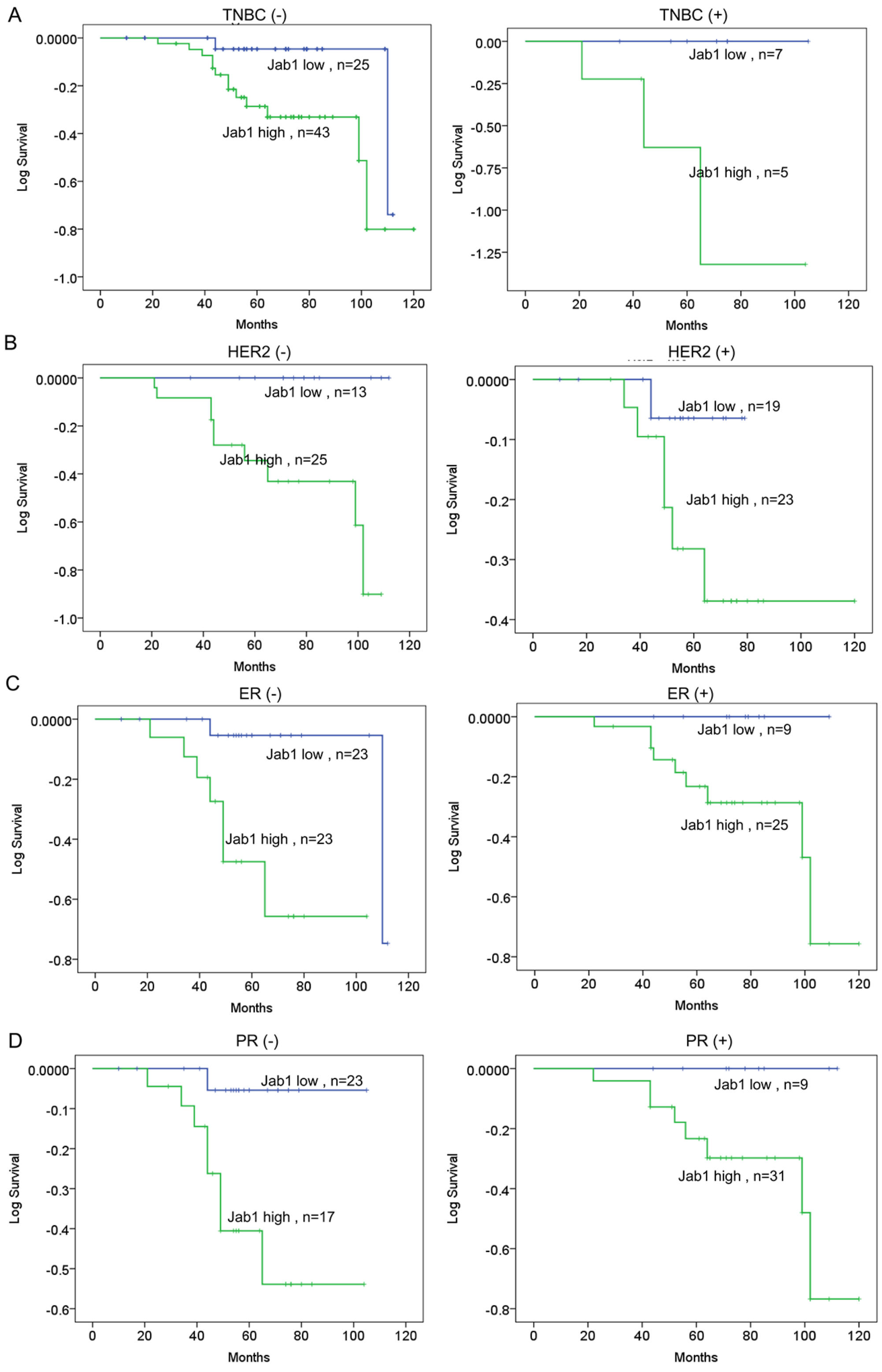

Supplementary Figure 2: Kaplan-Meier analyses of the association between Jab1 protein expression and survival in different subtype of breast cancer.
